# Supplementary material for: Ratiometric fluorescent sensing of pyrophosphate with sp³-functionalized single-walled carbon nanotubes
Source: Nat Commun. 2024 Jan 24;15:706. doi: 10.1038/s41467-024-45052-1 (PMC10808354; doi:10.1038/s41467-024-45052-1)
Supplement: Supplementary file 1 — Supplementary Information [file 41467_2024_45052_MOESM1_ESM.pdf]

SUPPLEMENTARY INFORMATION

Ratiometric fluorescent sensing of pyrophosphate with sp^3-functionalized single-walled carbon nanotubes

Simon Settele[1], C. Alexander Schrage[2], Sebastian Jung[2], Elena Michel[3], Han Li[4,5], Benjamin S. Flavel[4], A. Stephen K. Hashmi[3,6], Sebastian Kruss[2,7] and Jana Zaumseil[1]**

[1] Institute for Physical Chemistry, Universität Heidelberg, D-69120 Heidelberg, Germany

[2] Department of Chemistry and Biochemistry, Ruhr-Universität Bochum, D-44801 Bochum, Germany

[3] Institute for Organic Chemistry, Universität Heidelberg, D-69120 Heidelberg, Germany

[4] Institute of Nanotechnology, Karlsruhe Institute of Technology, Kaiserstrasse 12, D-76131 Karlsruhe, Germany

[5] Department of Mechanical and Materials Engineering, University of Turku, FI-20014 Turku, Finland

[6] Chemistry Department, Faculty of Science, King Abdulaziz University, 21589 Jeddah, Saudi Arabia

[7] Biomedical Nanosensors, Fraunhofer Institute for Microelectronic Circuits and Systems, D-47057 Duisburg, Germany

* corresponding authors: zaumseil@uni-heidelberg.de, sebastian.kruss@rub.de

CONTENTS

Supplementary Figures and Tables **3**
Suppl. Fig. 1 | Copper induced quenching of *sp*^{3}-functionalized (6,5) SWNTs 3
Suppl. Fig. 2 | Reversible quenching of *sp*^{3}-functionalized (6,5) SWNTs 3
Suppl. Fig. 3 | Effect of PP$_i$ on the PL of functionalized SDS-dispersed (6,5) SWNTs 4
Suppl. Note 1 | Effect of PP$_i$ on the PL of functionalized SDS-dispersed (6,5) SWNTs 4
Suppl. Fig. 4 | Dependence of PL area ratio and E$_{11}$ peak position on PP$_i$ concentration 5
Suppl. Fig. 5 | Sensor response in the presence of various analytes 5
Suppl. Table 1 | Extracted emission peak positions after addition of various analytes 6
Suppl. Fig. 6 | Sensing of different mixing ratios of PP$_i$ and ATP 7
Suppl. Fig. 7 | Impact of THPTA 7
Suppl. Fig. 8 | Competitive complexation of Cu^{2+} ions 8
Suppl. Note 2 | Competitive complexation of Cu^{2+} ions 8
Suppl. Fig. 9 | Impact of the functional group 9
Suppl. Fig. 10 | Comparison between functionalized and pristine (6,5) SWNTs 9
Suppl. Note 3 | Comparison between functionalized and pristine (6,5) SWNTs 9
Suppl. Fig. 11 | PP$_i$ Sensing with E$_{11}$* and E$_{11}$*$^-$ emission 10
Suppl. Table 2| Extracted values for PP$_i$ sensing with E$_{11}$* and E$_{11}$*$^-$ emission 10
Suppl. Fig. 12 | Impact of {Cu} on the absorption spectra 11
Suppl. Fig. 13 | Detection of PP$_i$ under pulsed laser excitation 11
Suppl. Table 3 | Extracted lifetime values from TCSPC measurements 12
Suppl. Fig. 14 | Investigation of the quenching mechanism 12
Suppl. Note 4 | Estimation of PET & mechanistic considerations 13
Suppl. Fig. 15 | Influence of the defect density on sensor sensitivity 14
Suppl. Note 5 | Influence of the defect density on sensor sensitivity 15
Suppl. Fig. 16 | Transfer of ethynylbenzene-functionalized (6,5) SWNTs to PL-PEG$_{5000}$ 16
Suppl. Fig. 17| Cu^{2+} ion induced quenching for DOC-coated (6,5) SWNTs 16
Suppl. Fig. 18| Effect of PP$_i$ on the PL of functionalized PL-PEG$_{5000}$-coated (6,5) SWNTs .. 17
Suppl. Fig. 19 | Detection of PP$_i$ in PL-PEG$_{5000}$ and buffer system 17
Suppl. Fig. 20 | Detection of PP$_i$ at high defect density 18
Suppl. Fig. 21 | Shift of PL peak positions at high defect density 19
Suppl. Fig. 22 | Comparison of sensing metrics at low and high defect densities 20
Suppl. Table 4 | Extracted dissociation constants and Hill-parameters 20
Suppl. Fig. 23 | Determination of the limit of detection 21
Suppl. Note 6 | Determination of the limit of detection 21
Suppl. Fig. 24 | Spiking experiment – shift in peak position 22
Suppl. Fig. 25| Sensing of PP$_i$ released in PCR 22
Suppl. Fig. 26 | Synthesis of arenediazonium tetrafluoroborates 23
Suppl. Note 7 | Synthesis of arenediazonium tetrafluoroborates 23
Suppl. Fig. 27 | Workflow sample preparation of functionalized (6,5) SWNTs dispersed in PL-PEG$_{5000}$ ready for PP$_i$ sensing 24
Supplementary References **25**

Supplementary Figures and Tables

Suppl. Fig. 1 | Copper induced quenching of *sp*^{3}-functionalized (6,5) SWNTs

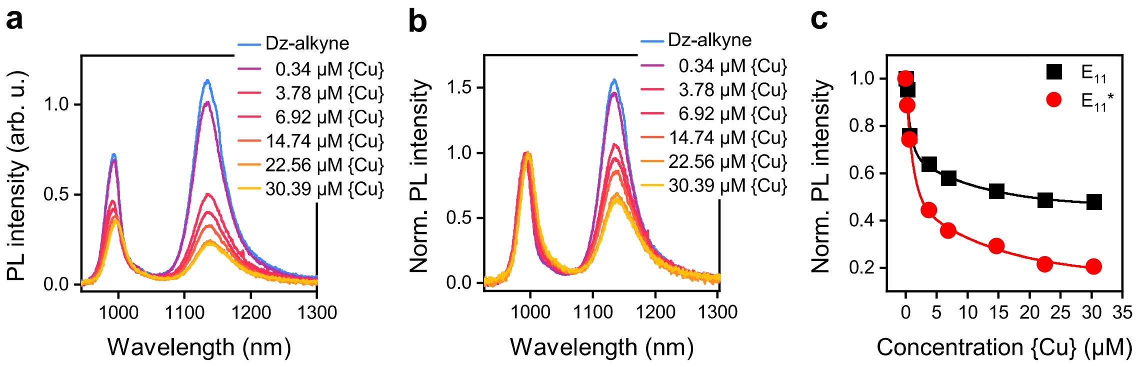

Supplementary Fig. 1 | Copper induced quenching. **a,b** Absolute (**a**) and normalized (**b**) PL spectra of 4-ethynylbenzene-functionalized (6,5) SWNTs after the addition of various concentration of {Cu}. **c** Norm. E_{11} (black) and E_{11}* (red) PL intensity *vs.* concentration of {Cu} (lines are guides to the eye). Source data are provided as a Source Data file.

Suppl. Fig. 2 | Reversible quenching of *sp*^{3}-functionalized (6,5) SWNTs

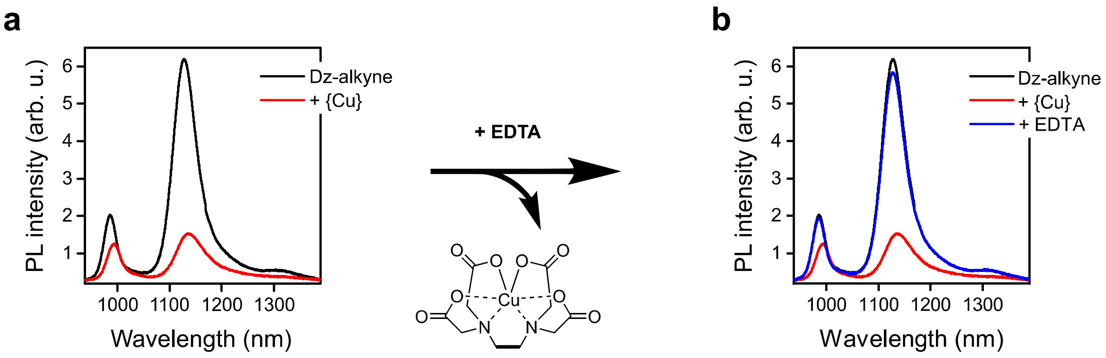

Supplementary Fig. 2 | Reversible quenching with EDTA. a PL spectra of 4-ethynylbenzene-functionalized (6,5) SWNTs before and after the addition of {Cu} (30 µM). **b** Further PL spectra after the addition of EDTA (1 mM). The expected complex formed by Cu^{2+} ions and EDTA is indicated below the black arrows. Source data are provided as a Source Data file.

Suppl. Fig. 3 | Effect of PP*ᵢ* on the PL of functionalized SDS-dispersed (6,5) SWNTs

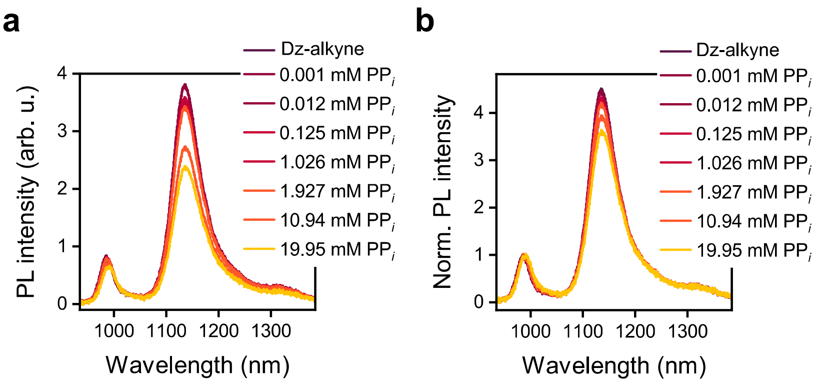

Supplementary Fig. 3. | Effect of PP$_i$ on SDS-dispersed (6,5) SWNTs. Absolute (**a**) and normalized (**b**) PL spectra of 4-ethynylbenzene-functionalized (6,5) SWNTs dispersed in SDS before and after the addition of various concentrations of PP$_i$. Source data are provided as a Source Data file.

Suppl. Note 1 | Effect of PP$_i$ on the PL of functionalized SDS-dispersed (6,5) SWNTs

For SDS-dispersed SWNTs, a small decrease in the overall PL intensity and the E_{11}*/E_{11} ratio can be observed for low concentrations of PP$_i$ (< 2 mM) and a significant drop at high concentrations of PP$_i$ (> 11 mM). The latter decrease in PL intensity is accompanied by a small red-shift of the E_{11} and E_{11}* emission peaks. Aggregation of surfactant-dispersed SWNTs is expected at high salt concentrations as described by Niyogi *et al.* and Koh *et al.* and could cause the observed drop in PL intensity as well as the red-shift.[1,2] However, the E_{11}*/E_{11} PL ratio is affected to a lesser extent.

Suppl. Fig. 4 | Dependence of PL area ratio and E_{11} peak position on PP_i concentration

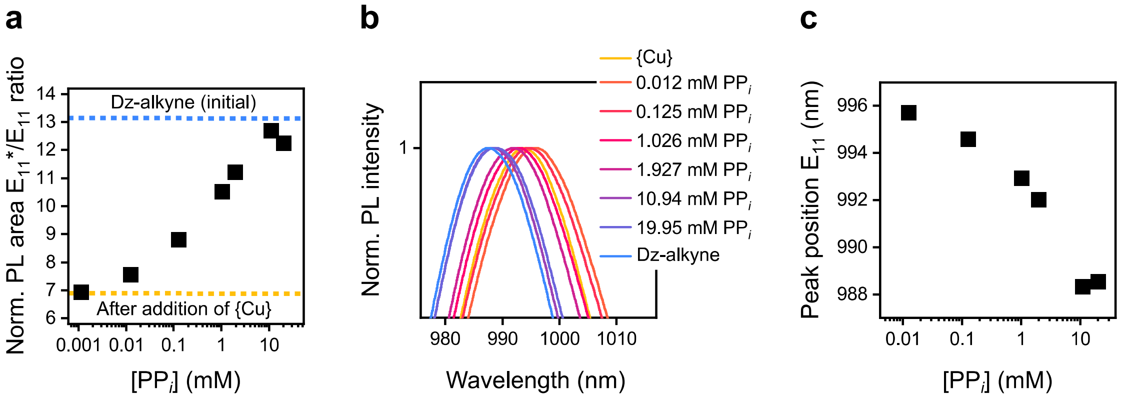

Supplementary Fig. 4. | PP_i dependent changes in PL characteristics. a E_{11}*/E_{11} PL area ratio upon addition of various concentrations of PP_i. Initial PL area ratio before and after the addition of {Cu} is indicated by blue and yellow dotted lines, respectively. **b** Zoom-in on the normalized E_{11} peak of ethynylbenzene-functionalized (6,5) SWNTs before and after the addition of {Cu} (15 µM) and various concentrations of PP_i. **c** E_{11} peak position upon addition of various concentrations of PP_i. Source data are provided as a Source Data file.

Suppl. Fig. 5 | Sensor response in the presence of various analytes

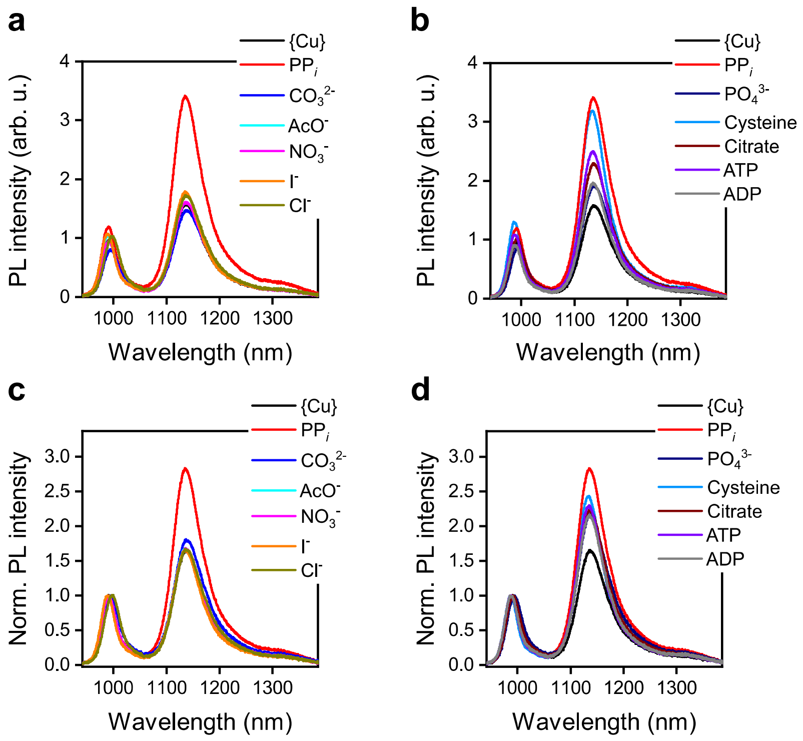

Supplementary Fig. 5. | Sensor response towards variouse analytes. a,c Response of the absolute (**a**) and normalized (**c**) PL of 4-ethynylbenzene-functionalized (6,5) SWNTs in the presence of various analytes that weakly complex copper(II) (1 mM). **b,d** Response of the absolute (**b**) and normalized (**d**) PL in the presence of various analytes which strongly complex copper(II) (1 mM). In all cases 15 µM {Cu} was used. Source data are provided as a Source Data file.

Suppl. Table 1 | Extracted emission peak positions after addition of various analytes

Supplementary Table 1. | Extracted peak position after addition of various analytes. E_{11} and E_{11}* peak position and optical trap depth Δ*E* after addition of various analytes (1 mM).

Sample/Analyte	Molecular structure	E_{11} (nm)	E_{11}* (nm)	Δ*E* (meV)
Dz-alkyne	-	986.2	1133.5	163.4
{Cu}	-	992.1	1137.7	159.9
PP_i	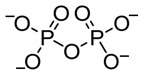	991.0	1135.8	159.5
ADP	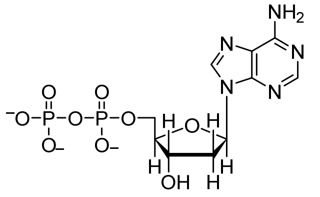	988.7	1135.8	162.4
ATP	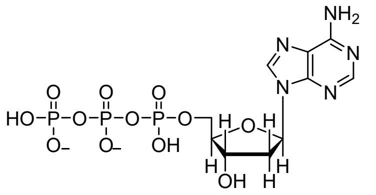	988.5	1135	161.9
L-Cysteine	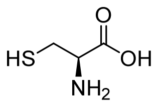	987.6	1133.6	161.7
Citrate	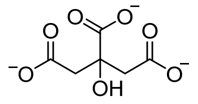	992.2	1136.3	158.5
I^-	-	989.6	1136.3	161.7
CO_3^{2-}	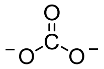	994.9	1138.1	156.8
AcO^-	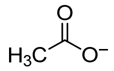	990.4	1136.3	160.7
NO_3^-	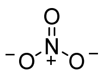	991.7	1137.4	160.1
PO_4^{3-}	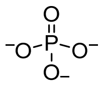	994.5	1136.3	155.6
Cl^-	-	997.9	1137.4	152.4

Suppl. Fig. 6 | Sensing of different mixing ratios of PP$_i$ and ATP

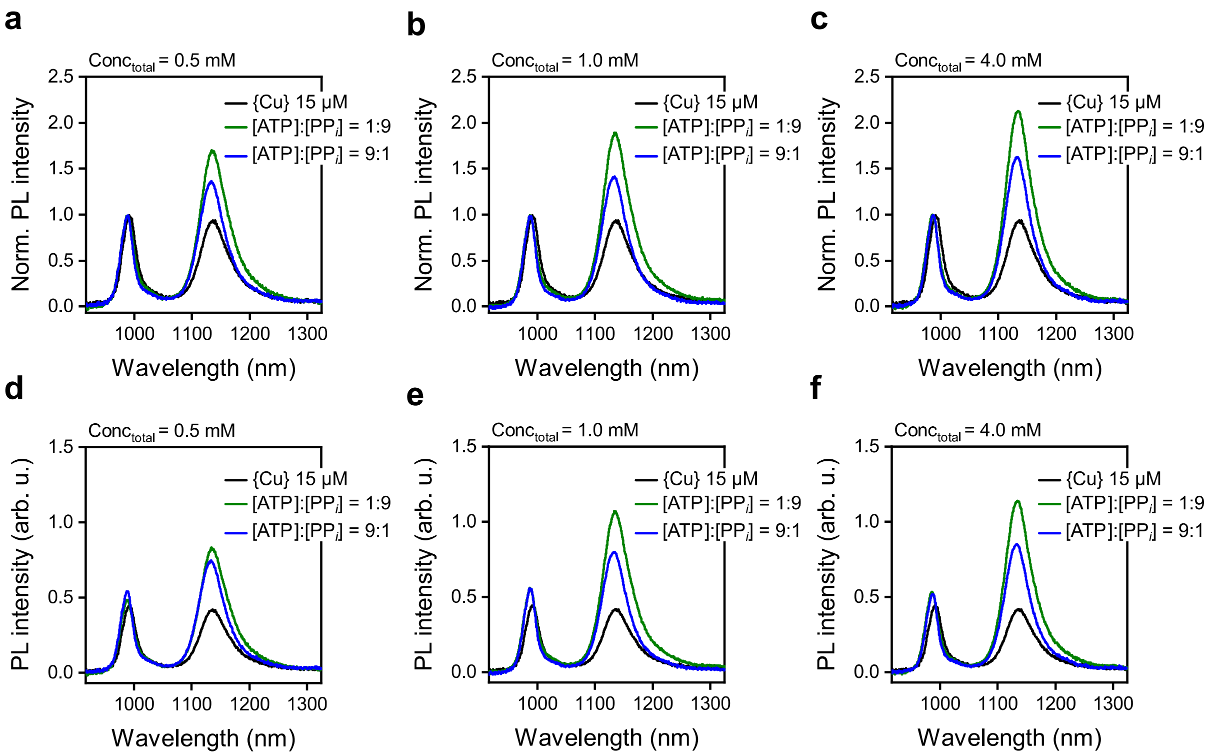

Supplementary Fig. 6. | Sensing of different mixing ratios. a-c Normalized PL spectra of 4-ethynylbenzene-functionalized (6,5) SWNTs after the addition of different ratios of ATP and PP$_i$ with a total analyte concentration of 0.5 mM, 1.0 mM and 4.0 mM. **d-f** Absolute PL spectra after the addition of different ratios of ATP and PP$_i$ with a total analyte concentration of 0.5 mM, 1.0 mM and 4.0 mM. In all cases 15 µM {Cu} was used. Source data are provided as a Source Data file.

Suppl. Fig. 7 | Impact of THPTA

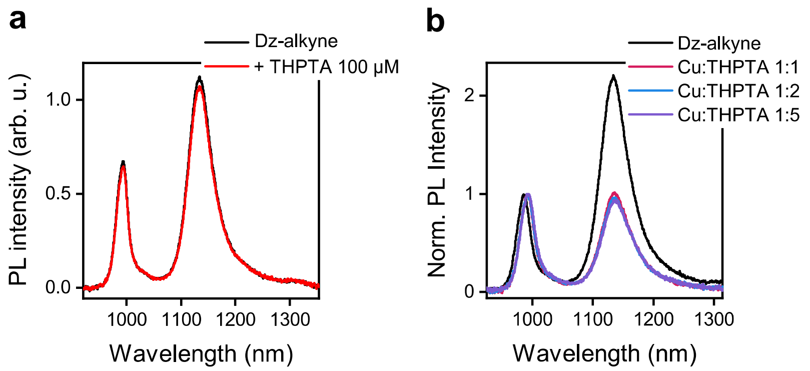

Supplementary Fig. 7. | Impact of THPTA. a PL Spectra of 4-ethynylbenzene-functionalized (6,5) SWNTs before and after the addition of 100 µM THPTA. **b** Normalized PL spectra after the addition of 15 µM {Cu} with different ratios of $CuSO_4(H_2O)_5$/THPTA. Source data are provided as a Source Data file.

Suppl. Fig. 8 | Competitive complexation of Cu^{2+} ions

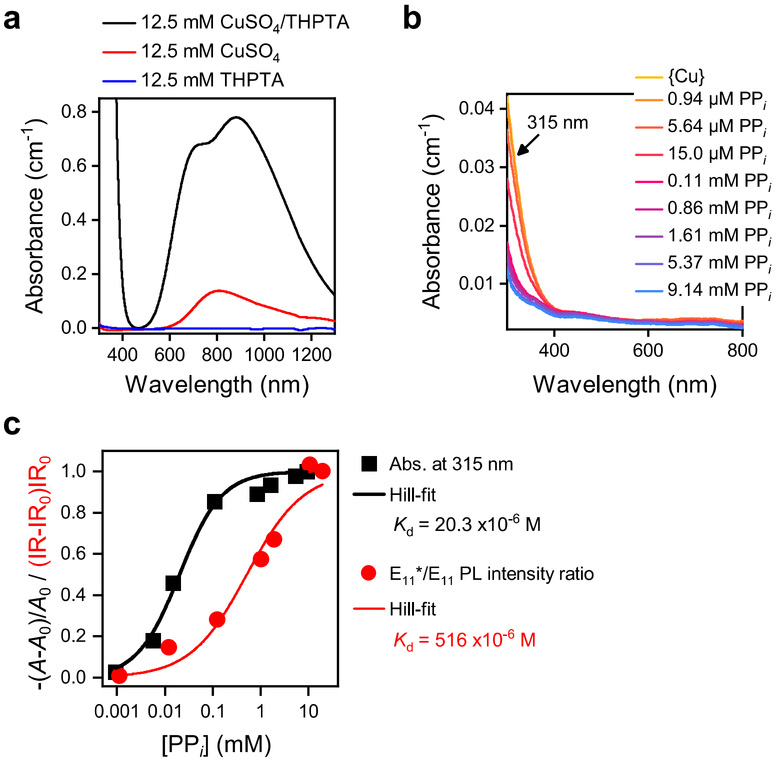

Supplementary Fig. 8. | Competitive compelxation of Cu^{2+} ions. a Absorption spectra of a 1:1 complex of $CuSO_4$ and THPTA (12.5 mM), $CuSO_4$ (12.5 mM) and THPTA (12.5 mM) **b** Absorption spectra of a 1:1 complex of $CuSO_4$ and THPTA (15 µM) after addition of various concentrations of PP_i. **c** Black squares: PP_i concentration dependent changes of absorption at 315 nm are displayed with A as the absorbance and A_0 as the absorbance before addition of PP_i. Red circles: PP_i concentration dependent changes of the E_{11}*/E_{11} PL intensity ratio extracted from PL measurements as displayed in Fig. 2. IR is the E_{11}*/E_{11} intensity ratio and IR_0 the E_{11}*/E_{11} intensity ratio after addition of {Cu}. The data were fitted with a Hill-function and dissociation constants of $20.3 \cdot 10^{-6}$ M and $516 \cdot 10^{-6}$ M were extracted, respectively. Source data are provided as a Source Data file.

Suppl. Note 2 | Competitive complexation of Cu^{2+} ions

The complexation/decomplexation of the Cu^{2+}/THPTA complex upon addition of PP_i can be tracked *in situ* by absorption spectroscopy. The absorption band around ≈315 nm is suitable for this purpose as it arises from the THPTA ligand when coordinated to $CuSO_4$. Neither the THPTA ligand itself nor $CuSO_4$ show significant absorption bands at this wavelength. Upon titration with PP_i, this absorption feature vanishes with increasing PP_i concentration indicating the removal of Cu^{2+} ions from the THPTA ligand. No absorption feature for the PP_i/Cu^{2+} complex is expected at this absorption wavelength due to the lack of an aromatic backbone. Hence, the concentration of Cu/THPTA complex can be directly correlated with the absorbance at 315 nm. When fitted with a Hill-function a dissociation constant of $K_d = 20.3 \cdot 10^{-6}$ M can be extracted. This value can be compared to the dissociation constant for the Cu^{2+}/THPTA complex on the SWNT surface when the E_{11}*/E_{11} intensity ratio of the SWNT probe is tracked.

The corresponding K_d extracted from SWNT PL measurements is 25 times higher ($K_d = 516 \cdot 10^{-6}$ M) and shows the additional stabilization of the complex by the SWNT surface and the ethynylbenzene group attached to the sp^3 defect.

Suppl. Fig. 9 | Impact of the functional group

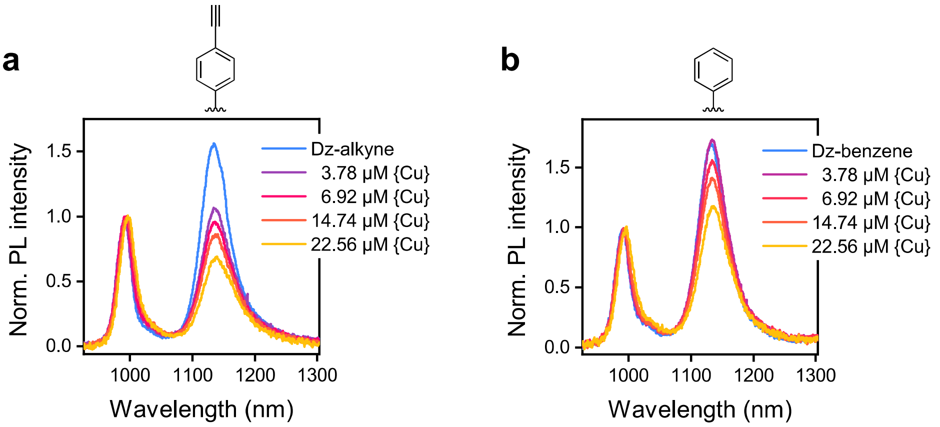

Supplementary Fig. 9. | Impact of the functional group. a Normalized PL spectrum of 4-ethynylbenzene-functionalized (6,5) SWNTs after the addition of various concentration of {Cu}. **b** Normalized PL spectra of benzene-functionalized (6,5) SWNTs after the addition of various concentration of {Cu}. Corresponding functional groups are indicated above the spectra. Source data are provided as a Source Data file.

Suppl. Fig. 10 | Comparison between functionalized and pristine (6,5) SWNTs

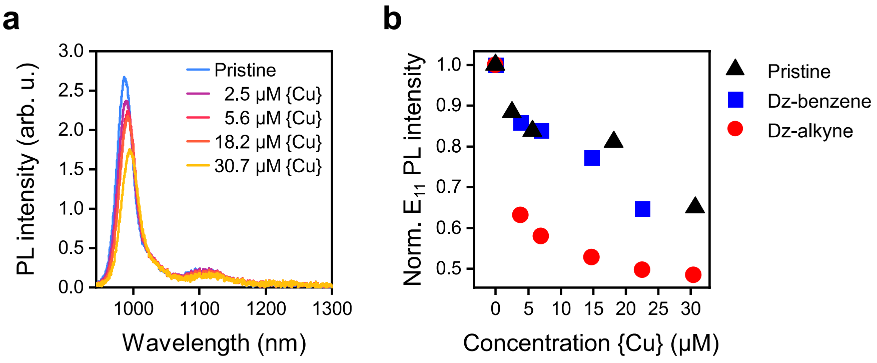

Supplementary Fig. 10. | Comparison in PL quenching. a PL spectrum of pristine (6,5) SWNTs after the addition of various concentrations of {Cu}. **b** Extracted E_{11} PL intensities normalized to their initial values before addition of {Cu} for pristine, benzene-functionalized and 4-ethnylbenzene-functionalized (6,5) SWNTs *vs.* concentration of {Cu}. Source data are provided as a Source Data file.

Suppl. Note 3 | Comparison between functionalized and pristine (6,5) SWNTs

PL quenching after addition of {Cu} occurs for pristine (6,5) SWNTs to a similar degree as observed for functionalized SWNTs with a benzene moiety. Thus, we can consider quenching of E_{11} excitons to be independent of $E_{11}{}^*$ excitons. The stronger quenching effect for

functionalized SWNTs with alkynyl moiety is likely to originate from additional coordination of {Cu} to the alkynyl group, which facilitates adsorption to the SWNT surface.

Suppl. Fig. 11 | PP$_i$ Sensing with E_{11}* and E_{11}*$^{-}$ emission

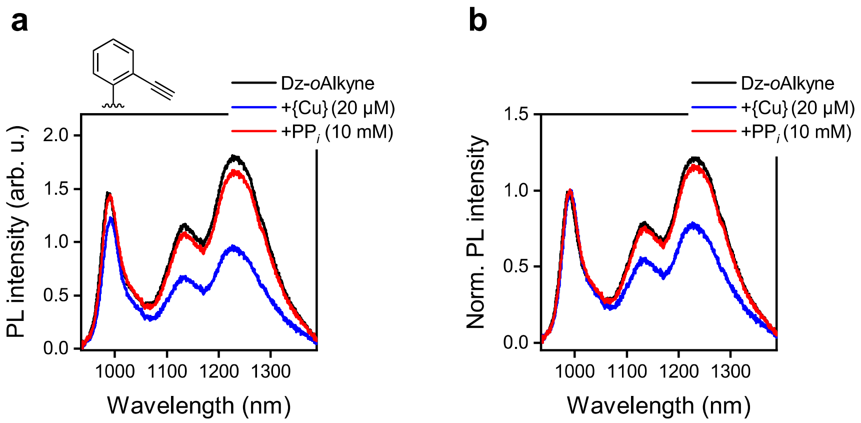

Supplementary Fig. 11. | PP$_i$ sensing with E_{11}* and E_{11}*$^{-}$ emission. a,b Absolute (**a**) and normalized (**b**) PL spectra of 2-ethynylbenzene-functionalized (*o*Alkyne) (6,5) SWNTs after the addition of 20 µM {Cu}. The functional group is indicated above the spectrum in **a**. Source data are provided as a Source Data file.

Suppl. Table 2| Extracted values for PP$_i$ sensing with E_{11}* and E_{11}*$^{-}$ emission

Supplementary Table 2. | Extracted values for PP$_i$ sensing. Extracted peak positions, area ratios and quenching factors (QF) for E_{11}*$^{-}$ and E_{11}*$^{-}$ emission after fitting with Voigt functions. Note that E_{11}*$^{-}$ emission is quenched stronger than E_{11}* emission, likely caused by its initially longer PL lifetime.

Sample	Dz-*o*Alkyne	+{Cu}
Peak position E_{11} (nm)	990	995
Peak position E_{11}* (nm)	1128	1127
Peak position E_{11}*$^{-}$ (nm)	1242	1240
Area ratio E_{11}*/E_{11}	1.00	0.86
Area ratio E_{11}*$^{-}$/E_{11}	2.93	2.13
QF E_{11}*/E_{11} (area ratio)	-	1.16
QF E_{11}*$^{-}$/E_{11} (area ratio)	-	1.38

Suppl. Fig. 12 | Impact of {Cu} on the absorption spectra

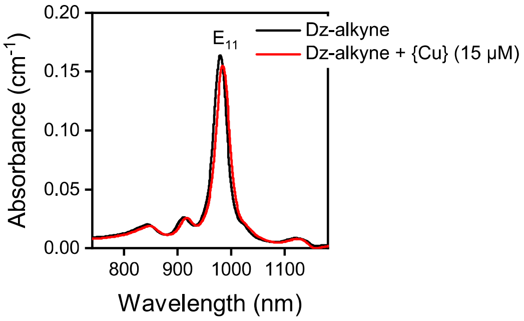

Supplementary Fig. 12. | Imapct of {Cu} on the absorption spectra. Absorption spectra of 4-ethynylbenzene-functionalized (6,5) SWNTs before and after addition of 15µM {Cu}. Source data are provided as a Source Data file.

Suppl. Fig. 13 | Detection of PP*i* under pulsed laser excitation

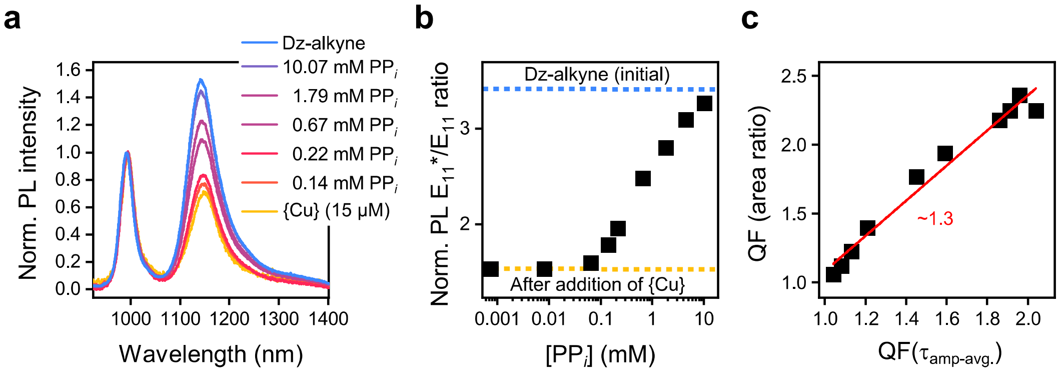

Supplementary Fig. 13. | Detection of PP*i* under pulsed laser excitation. a Normalized PL spectrum under pulsed laser excitation (as in TCSPC measurements for data in Table S3) after addition of various concentrations of PP*i*. **b** E_{11}*/E_{11} PL area ratio upon addition of various concentrations of PP*i*. The initial PL area ratios before and after the addition of {Cu} are indicated by blue and yellow dotted lines, respectively. **c** QF extracted from E_{11}/E_{11}* PL area ratios plotted *vs*. the QFs extracted from amplitude averaged lifetimes ($\tau_{\text{amp-avg.}}$) obtained by TCSPC measurements (see Supplementary Table 3). The red solid line is a linear fit to the data with a slope of ~1.3. The strong correlation between QFs extracted from PL area ratios and $\tau_{\text{amp-avg.}}$ indicates the absence of ground-state quenching. Source data are provided as a Source Data file.

Suppl. Table 3 | Extracted lifetime values from TCSPC measurements

Supplementary Table 3. | Extracted lifetime values from TCSPC. Extracted $\tau_{\text{amp-avg.}}$ from TCSPC measurements and $E_{11}/E_{11}*$ PL area ratios with corresponding QF.

Sample	$\tau_{\text{amp-avg.}}$	QF ($\tau_{\text{amp-avg.}}$)	Area ratio	QF (Area ratio)
Dz-alkyne	151		3.47	
+{Cu}	79	1.91	1.54	2.25
0.75 µM	74	2.04	1.54	2.25
8.27 µM	77	1.96	1.47	2.36
64.7 µM	81	1.86	1.59	2.18
0.14 mM	95	1.59	1.79	1.94
0.22 mM	104	1.45	1.96	1.77
0.67 mM	125	1.21	2.48	1.40
1.80 mM	134	1.13	2.81	1.23
4.43 mM	140	1.08	3.10	1.12
10.07 mM	145	1.04	3.27	1.06

Suppl. Fig. 14 | Investigation of the quenching mechanism

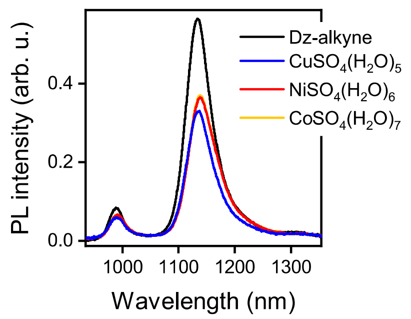

Supplementary Fig. 14. | Metal ion dependent PL quenching. PL spectra after addition of $CuSO_4(H_2O)_5$, $NiSO_4(H_2O)_6$ and $CoSO_4(H_2O)_7$ (0.25 mM). Source data are provided as a Source Data file.

Suppl. Note 4 | Estimation of PET & mechanistic considerations

Quenching mechanisms from paramagnetic metals are frequently expected to result from fast electron transfer mechanisms such as photoinduced electron transfer (PET) from the excited state of the fluorophore to the paramagnetic metal centre.[3-5] To evaluate this possibility, the Gibbs free energy of the PET was estimated according to

$$\Delta G_{\mathrm{PET}} = \mathrm{e}E_{\mathrm{red}}(D^{+}/D) - \mathrm{e}E_{\mathrm{red}}(A/A^{-}) - \Delta G_{00} - \frac{\mathrm{e}^2}{4\pi\varepsilon_{\mathrm{r}}\varepsilon_0 d} \quad (1)$$

where E_{red} is the reduction potential, ΔG_{00} the optical transition energy, ε_{r} the relative permittivity of water (ε_{r} (20 °C) = 80). A distance d of approximately 1.0 nm between the defect site and the metal centre was assumed. For the redox potential of functionalized (6,5) SWNTs (Donor, D) values reported by Shiraishi *et al.* were used (E_{ox} (V) = 0.584 *vs.* Ag/AgCl)[6] and for Cu(II)/Cu(I) (Acceptor, A) E_0 (V) = 0.161 was used.[7] Note, that the reduction potential of Cu(II) is expected to shift upon complexation with THPTA.[8] However, no exact values are reported and its redox potential may further vary upon adsorption to the SWNT sidewall. Hence, the effect of the ligand was not considered within this estimation. With an optical transition energy of ΔG_{00} = 1.09 eV, a large negative ΔG_{PET} was calculated (ΔG_{PET} = -2.05 eV). Thus, PET would be thermodynamically highly favourable and would present a viable route to quench E_{11}* emission (as well E_{11} emission).[9]

If the above assumption is correct, the quenching efficiency is expected to correlate with the standard reduction potential of the metal ion. With a standard reduction potential for Ni(II/0) and Co(II/0) of -0.26 V and -0.28 V, respectively, a negative ΔG_{PET} of -1.61 eV and -1.65 eV can be calculated.[7] Thus, PL quenching of Ni(II) and Co(II) *via* PET is also likely and quenching should occur to a similar degree. This notion agrees with our observation that Ni(II) and Co(II) exhibit similarly strong quenching (see Supplementary Fig. 11) while stronger quenching is observed for Cu(II). Other effects such as the influence of the counter ion and impact of pH value were previously reported to be negligible and thus were not further investigated within the scope of this work.[10] While our observations indicate PL quenching *via* PET, further studies with different metal ions are necessary to obtain a more conclusive picture.

Suppl. Fig. 15 | Influence of the defect density on sensor sensitivity

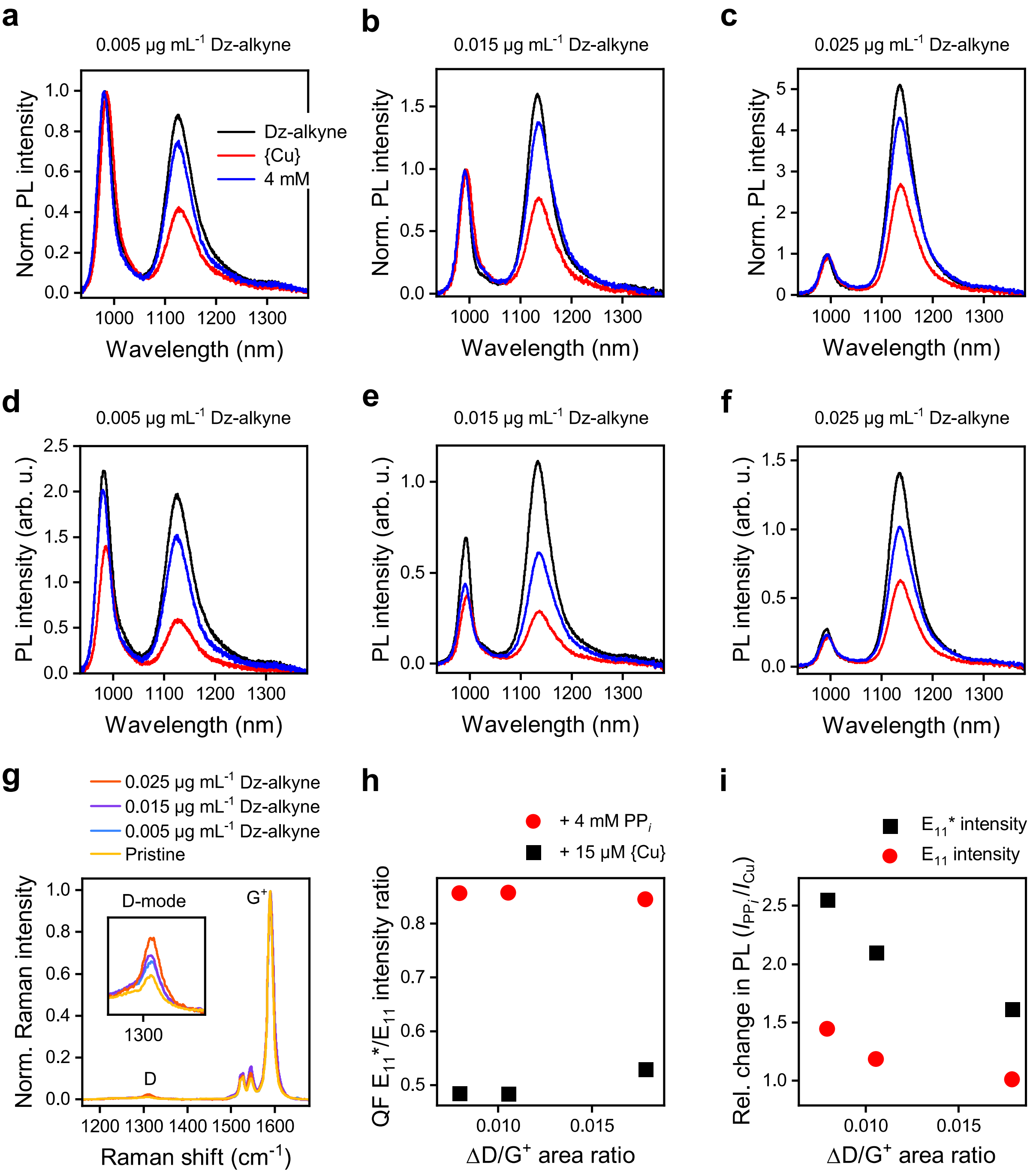

Supplementary Fig. 15. | Imapct of the defect density on PP*ᵢ* sensing. Normalized (**a-c**) and absolute (**d-f**) PL spectra of (6,5) SWNTs in SDS functionalized at various concentrations of 4-ethynyl benzene diazonium tetrafluoroborate after addition of 15 µM {Cu} and 4 mM PP_i. **g** Normalized Raman spectra of functionalized (6,5) SWNTs. **h** QF of the E_{11}*/E_{11} intensity ratio *vs.* change in D/G^+ area ratio (ΔD/G^+). **i** Relative changes in PL intensity (I_{PPi}/I_{Cu}) of E_{11} and E_{11}* emission *vs.* change in D/G^+ area ratio (ΔD/G^+). Source data are provided as a Source Data file.

Suppl. Note 5 | Influence of the defect density on sensor sensitivity

PL and Raman spectra were recorded for SWNT dispersions with three different defect densities that were obtained by variation of the 4-ethynylbenzene diazonium tetrafluoroborate concentration during functionalization. Normalized as well as absolute PL spectra and Raman spectra are shown in Supplementary Fig. 15. The defect density after functionalization correlates with the differential Raman $\Delta D/G^{+}$ area ratio, representing the change in D/G^{+} area ratio compared to the pristine sample. Upon addition of 15 µM {Cu} similarly strong quenching and PL recovery after addition of 4 mM PP_i is observed, although, with subtle differences. Extracting the QF ($I_{\{Cu\}}/I_{Ref\text{-}alkyne}$) of the PL E_{11}*/E_{11} intensity ratio and plotting it against the $\Delta D/G^{+}$ area ratio shows that the overall reduction of the PL ratio is slightly reduced at higher defect densities. This is reasonable, as more defect sites must be coordinated by Cu^{2+} ions in contrast to samples with low defect densities to achieve a similarly strong quenching of the defect state emission. However, we do not find a change in the sensitivity/dynamic range for the PL E_{11}*/E_{11} intensity ratio. This observation agrees with data presented later (see Supplementary Fig. 22a).

In contrast to this, a dependence on the absolute PL intensities can be observed. When extracting the relative increase in PL intensities for E_{11} and E_{11}* emission, *i.e.* the ratio of PL intensity before and after the addition of PP_i ($I_{PPi}/I_{\{Cu\}}$) (see Supplementary Fig. 15i), a clear correlation with the defect density is found. In this case, a higher sensitivity is obtained for low defect densities. Supplementary Fig. 22b shows a similar effect, where the dynamic range shifts toward higher concentrations of PP_i for SWNTs that were functionalized to a higher degree.

In summary, the best sensitivity can be obtained at very low defect densities, however, its impact on the PL intensity ratio is minimal. Thus, we recommend to use defect densities that are close or slightly below the PLQY maximum of functionalized SWNTs to obtain an overall high PL signal with good sensitivity.

Suppl. Fig. 16 | Transfer of ethynylbenzene-functionalized (6,5) SWNTs to PL-PEG$_{5000}$

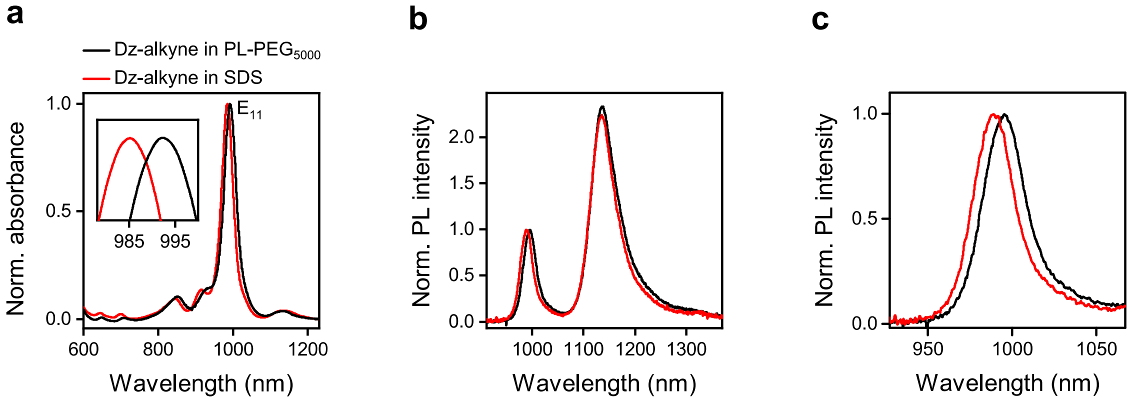

Supplementary Fig. 16. | Surfactant exchange to PL-PEG$_{5000}$. **a** Normalized absorption spectrum before and after transfer to PL-PEG$_{5000}$ with zoom-in on the E_{11} transition. **b** Normalized PL spectra before and after transfer to PL-PEG$_{500}$. **c** Zoom-in on E_{11} PL peak. Source data are provided as a Source Data file.

Suppl. Fig. 17| Cu^{2+} ion induced quenching for DOC-coated (6,5) SWNTs

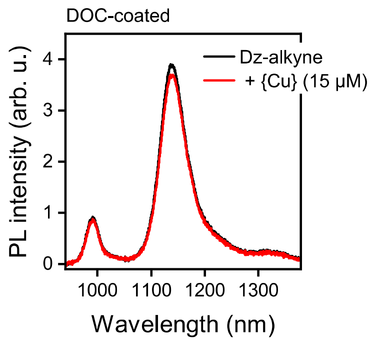

Supplementary Fig. 17. | Copper induced quenching for DOC-coated (6,5) SWNTs. PL spectra of 4-ethynylbenzene-functionalized (6,5) SWNTs dispersed in DOC before and after addition of 15 µM {Cu}. Source data are provided as a Source Data file.

Suppl. Fig. 18| Effect of PP$_i$ on the PL of functionalized PL-PEG$_{5000}$-coated (6,5) SWNTs

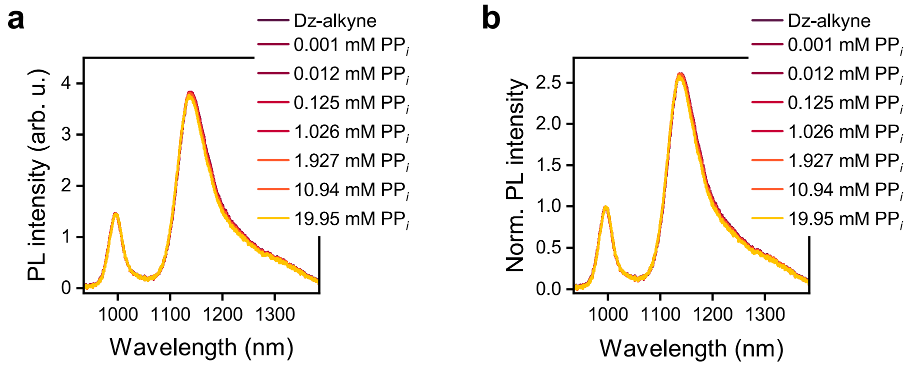

Supplementary Fig. 18. | Effect of PP$_i$ on PL-PEG$_{5000}$ dispersed (6,5) SWNTs. Absolute (**a**) and normalized (**b**) PL spectra of 4-ethynylbenzene-functionalized (6,5) SWNTs dispersed in PL-PEG$_{5000}$ before and after the addition of various concentrations of PP$_i$. Source data are provided as a Source Data file.

Suppl. Fig. 19 | Detection of PP$_i$ in PL-PEG$_{5000}$ and buffer system

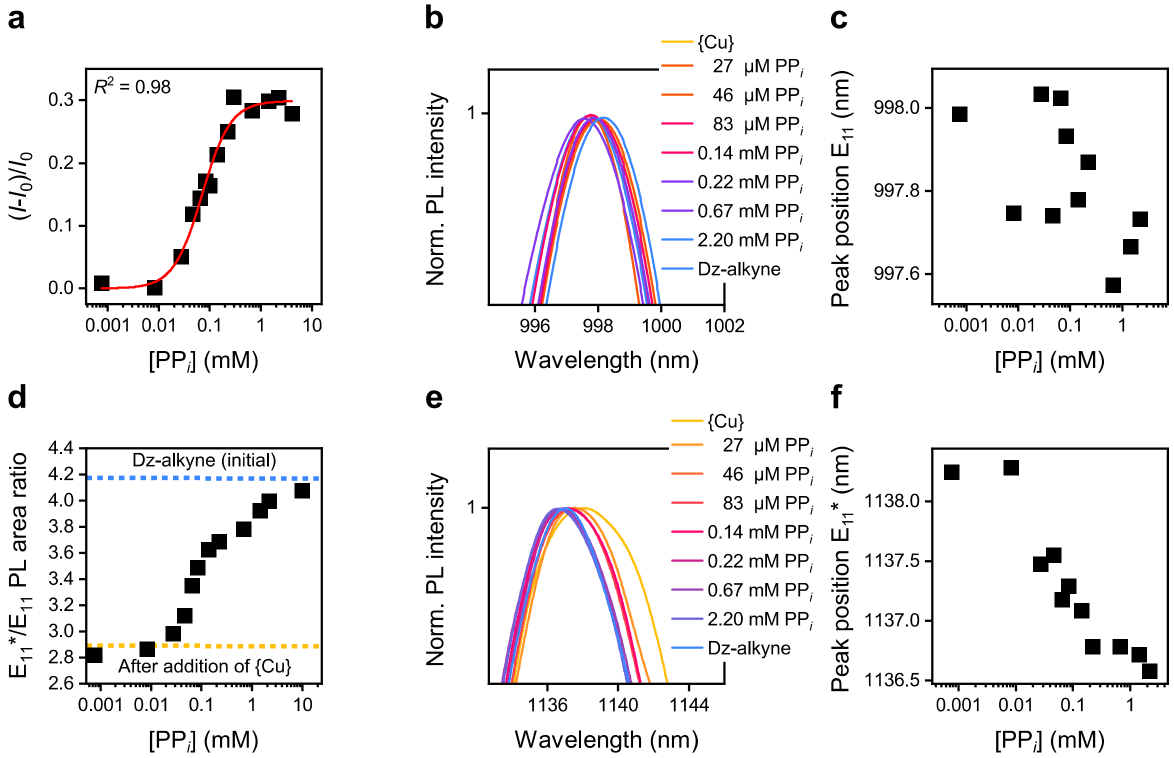

Supplementary Fig. 19. | Detection of PP$_i$ in buffer system. a,d PP$_i$ concentration dependent changes of E$_{11}$ PL intensities (**a**) and PL area ratios (**d**). Initial E$_{11}$*/E$_{11}$ PL area ratio before and after the addition of {Cu} is indicated by blue and yellow dotted lines, respectively. Red line shows the fitted curve for $Y = [PP_i]^n/(K_d{}^n + [PP_i]^n)$. The coefficient of determination (R^2) of the fit is given in the plot. **b,e** Zoom-in on the normalized E$_{11}$ (**b**) and E$_{11}$* (**e**) peak at various concentrations of PP$_i$. **c,f** E$_{11}$ (**c**) and E$_{11}$* (**f**) peak position *vs*. PP$_i$ concentration. Source data are provided as a Source Data file.

Suppl. Fig. 20 | Detection of PP*ᵢ* at high defect density

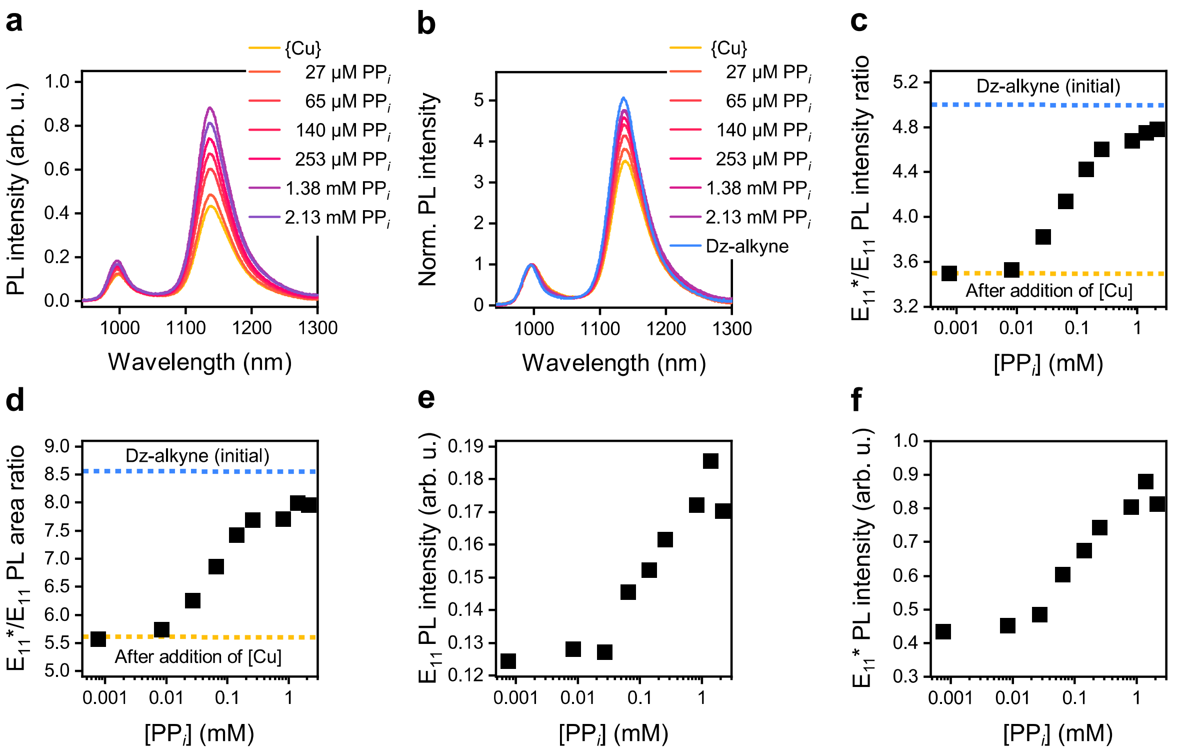

Supplementary Fig. 20. | Detection of PP*ᵢ* at high defec density. a,b Absolute (**a**) and normalized (**b**) PL spectra of 4-ethynylbenzene-functionalized (6,5) SWNTs in PL-PEG$_{5000}$ and 10 mM MOPS buffer at various concentrations of PP$_i$. **c** PL intensity ratio *vs.* PP$_i$ concentration. **d** PL area ratio *vs.* PP$_i$ concentration. **e** E$_{11}$ PL intensity *vs.* PP$_i$ concentration. **f** E$_{11}$* PL intensity *vs.* PP$_i$ concentration. Initial E$_{11}$*/E$_{11}$ PL intensity and area ratios before and after the addition of {Cu} are indicated by blue and yellow dotted lines, respectively. Source data are provided as a Source Data file.

Suppl. Fig. 21 | Shift of PL peak positions at high defect density

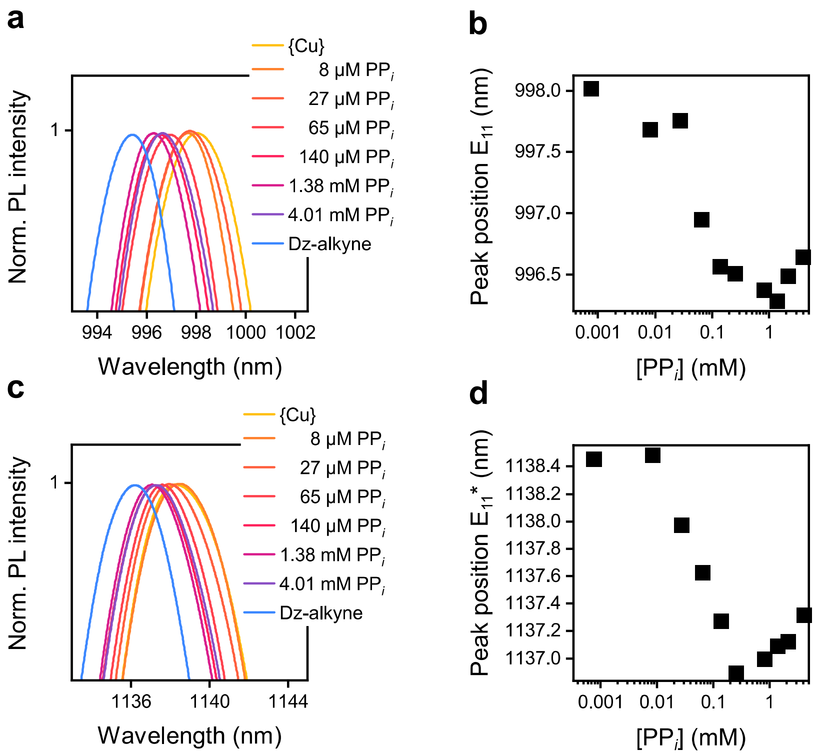

Supplementary Fig. 21. | Shift of PL peak position. a,c Zoom-in on the normalized E_{11} and E_{11}* peak at various concentrations of PP$_i$. **b,d** E_{11} and E_{11}* peak position *vs*. PP$_i$ concentration. Source data are provided as a Source Data file.

Suppl. Fig. 22 | Comparison of sensing metrics at low and high defect densities

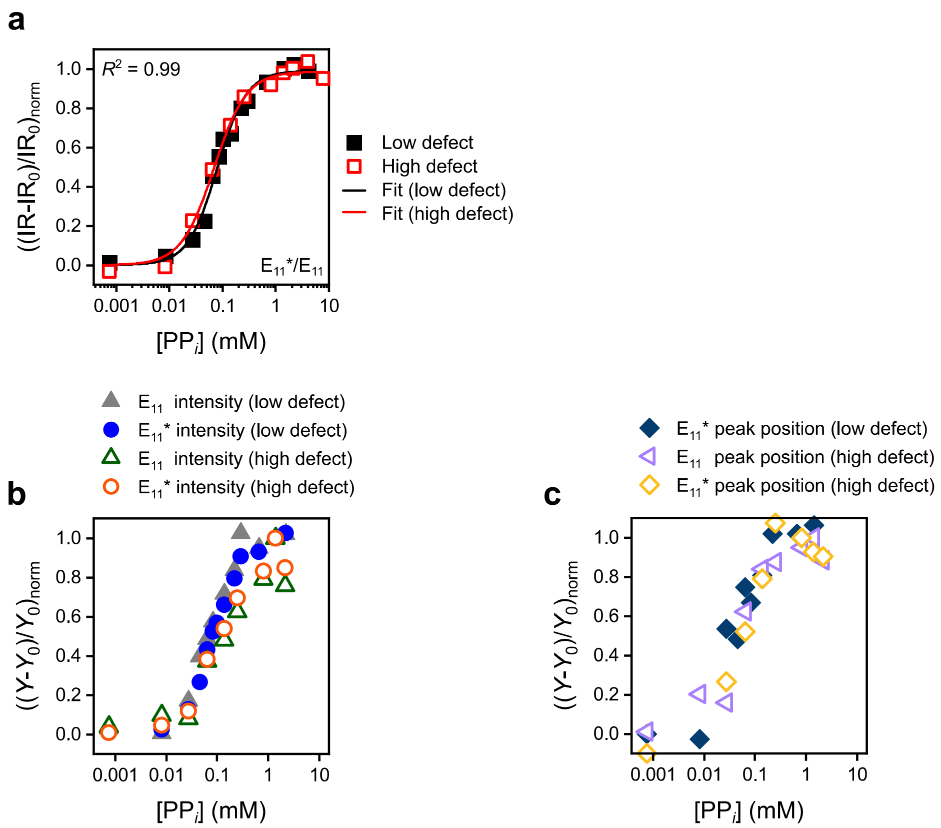

Supplementary Fig. 22. | Comparisson of sensing metrics. a PP_i concentration dependent changes of the normalized E_{11}*/E_{11} PL intensity ratio for high and low sp^3 defect densities. Lines show the fitted curves for $Y = [PP_i]^n/(K_d^n + [PP_i]^n)$, with IR - normalized E_{11}*/E_{11} intensity ratio and IR_0 – normalized E_{11}*/E_{11} intensity ratio after the addition of {Cu}. **b,c** PP_i concentration dependent changes of the normalized E_{11}* and E_{11} PL intensities (**b**) and E_{11}* and E_{11} peak positions (**c**) for high and low and high sp^3 defect densities, with Y – normalized PL intensity and peak position and Y_0 – normalized PL intensity and peak position after the addition of {Cu}. For changes in E_{11} peak position at low defect density please see Fig. S13c. Source data are provided as a Source Data file.

Suppl. Table 4 | Extracted dissociation constants and Hill-parameters

Supplementary Table 4. | Extracted parameters from Hill-model. Dissociation constant (K_d) and Hill-parameter (n) for normalized E_{11}*/E_{11} PL intensity ratios fit with the Hill-model.

Sample	K_d (10^{-6} M)	n
low sp^3 defect concentration	79.3	1.57
high sp^3 defect concentration	68.4	1.42

Suppl. Fig. 23 | Determination of the limit of detection

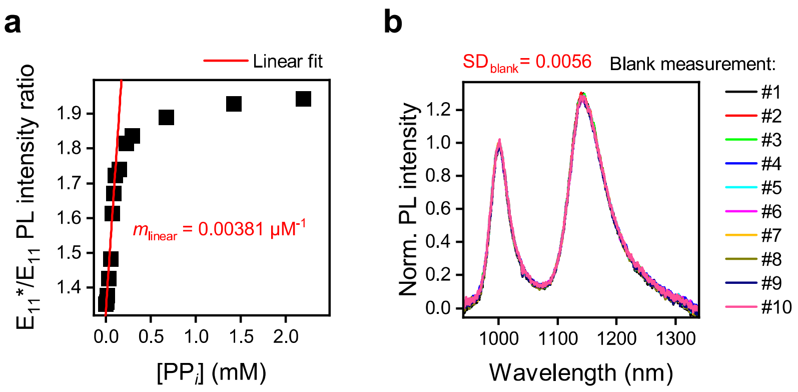

Supplementary Fig. 23. | Determination of the LOD. a PP$_i$ concentration dependent changes of the E_{11}^*/E_{11} PL intensity ratio with linear fit to data at low concentrations of PP$_i$ (m_{linear} = 0.00381 µM^{-1}). b Normalized PL spectra of 4-ethynylbenzene-functionalized (6,5) SWNTs dispersed in PL-PEG$_{5000}$ and 10 mM MOPS buffer after addition of {Cu} with a standard deviation of the E_{11}^*/E_{11} PL intensity ratio of SD_{blank} = 0.0056 (number of blank measurements, n = 10). The displayed data correspond to the data set shown in Fig. 5. Source data are provided as a Source Data file.

Suppl. Note 6 | Determination of the limit of detection

The limit of detection (LOD) was calculated based on the standard deviation of the response upon addition of {Cu} to the SWNT sensor (SD_{blank}; n = 10) and the slope m_{linear} from a linear fit of the calibration curve at low PP$_i$ concentrations. The values for m_{linear} and SD_{blank} were extracted and measured from the same sample as shown in Fig.5.

$$\mathrm{LOD} = (3 \cdot \mathrm{SD_{blank}})/m_{\mathrm{linear}} \quad (2)$$

This analysis yielded a LOD for the different parameters of:

- E_{11} intensity: LOD = 5.2 µM
- E_{11}^* intensity: LOD = 12.5 µM
- E_{11}^*/E_{11} PL ratio: LOD = 4.4 µM

Suppl. Fig. 24 | Spiking experiment – shift in peak position

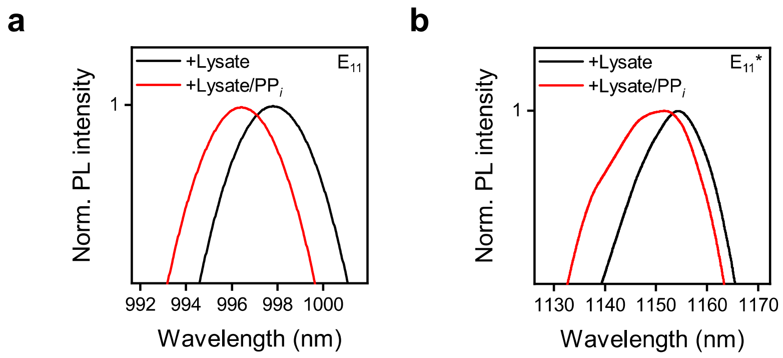

Supplementary Fig. 24. | Shift in PL peak position. a,b E_{11} (**a**) and E_{11}* (**b**) peak position of 4-ethynylbenzene-functionalized (6,5) SWNTs in PL-PEG$_{5000}$ quenched by {Cu} after addition of 0.5 µL cell-lysate which was subsequently spiked with 188 µM of PP$_i$. Source data are provided as a Source Data file.

Suppl. Fig. 25| Sensing of PP$_i$ released in PCR

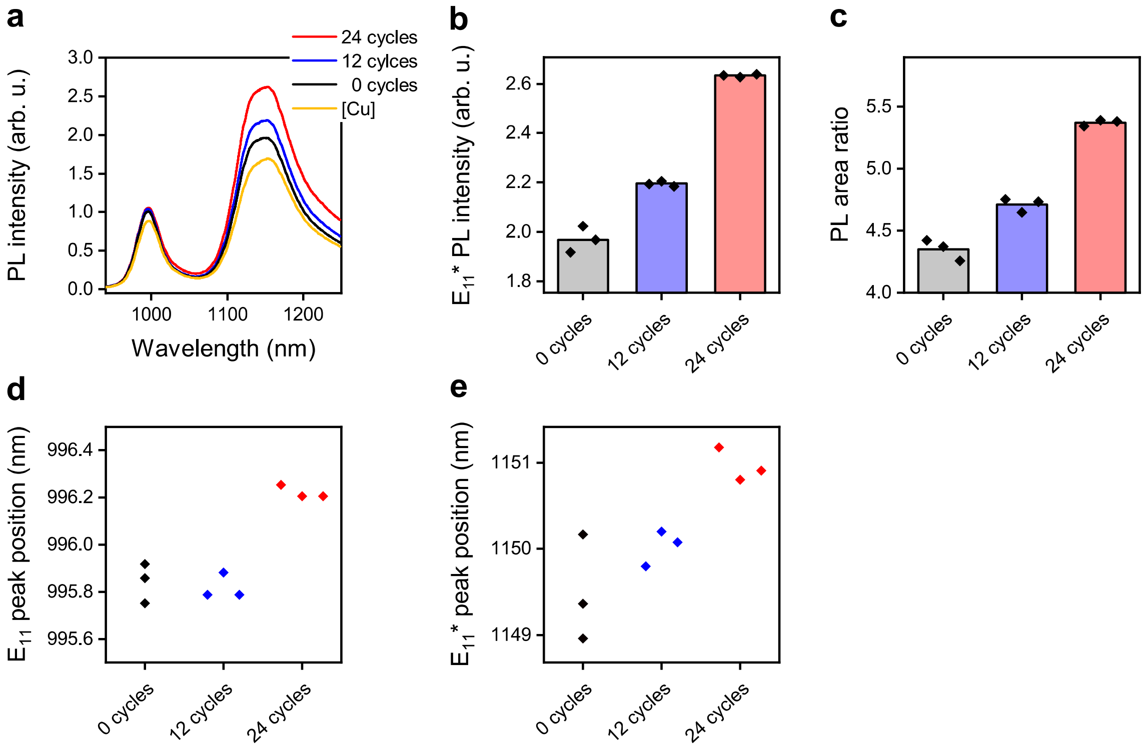

Supplementary Fig. 25. | Sensing of PP$_i$ released in PCR. a-e PL spectra (**a**, averaged over 3 measurements), absolute E_{11}* PL intensities (**b**), E_{11}*/ E_{11} PL area ratios (**c**), E_{11} peak positions (**d**) and E_{11}* peak positions (**e**) of 4-ethynylbenzene-functionalized (6,5) SWNTs dispersed in PL-PEG$_{5000}$ after the addition of 5 µL of PCR product after 0 (black/grey), 12 (blue) and 24 (red) cycles. One biologically independent sample was examined over n = 3 independent measurements with the SWNT sensor, and should represent the spread in the response of the sensor. Individual data points are indicated as diamonds. The height of the bar represents the average of the three measurements. Source data are provided as a Source Data file.

Suppl. Fig. 26 | Synthesis of arenediazonium tetrafluoroborates

Arenediazonium tetrafluoroborates were synthesized as described in the Methods sections. A similar procedure can be found in reference 11.[11]

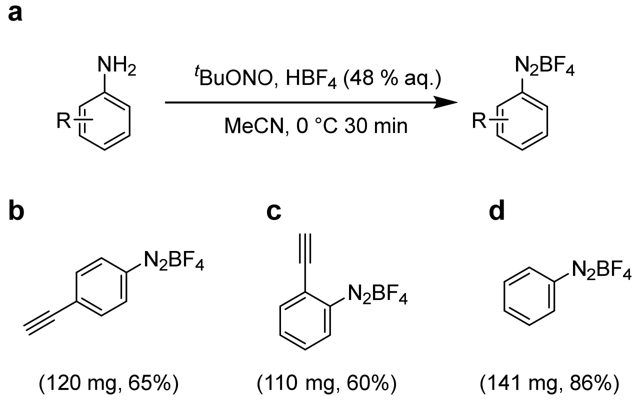

Supplementary Fig. 26 | Synthesis of diazonium salts. a Reaction scheme for the synthesis of diazonium salts. Preparation and obtained yield of 4-ethynylbenzene diazonium tetrafluoroborate (**b**) 2-ethynylbenzene diazonium tetrafluoroborate (**c**) and Benzene diazonium tetrafluoroborate (**d**). Source data of ^{1}H NMR are provided as a Source Data file.

Suppl. Note 7 | Synthesis of arenediazonium tetrafluoroborates

^{1}H NMR of arenediazonium tetrafluoroborates

4-ethynylbenzene diazonium tetrafluoroborate:

^{1}H NMR (400 MHz, CD_3CN): δ = 8.45 (d, J = 8.8 Hz, 2H), 7.96 (d, J = 8.8 Hz, 2H), 4.23 (s, 1H) ppm. The data are consistent with the previous literature.[12]

2-ethynylbenzene diazonium tetrafluoroborate:

^{1}H NMR (400 MHz, $(CD_3)_2SO$): δ = 8.81 (dd, J = 8.4, 1.1 Hz, 1H), 8.28 (td, J = 7.8, 1.2 Hz, 1H), 8.16 (dd, J = 7.9, 1.0 Hz, 1H), 8.05-8.00 (m, 1H), 5.61 (s, 1 H) ppm. The data are consistent with the previous literature.[13]

Benzene diazonium tetrafluoroborate:

^{1}H NMR (400 MHz, $(CD_3)_2SO$): δ = 8.67 (d, J = 8.8, 1.2 Hz, 2H), 8.26 (t, J = 7.7 Hz, 1H), 7.98 (dd, J = 8.7, 7.7 Hz, 2H) ppm. The data are consistent with the previous literature.[14]

Suppl. Fig. 27 | Workflow sample preparation of functionalized (6,5) SWNTs dispersed in PL-PEG$_{5000}$ ready for PP$_i$ sensing

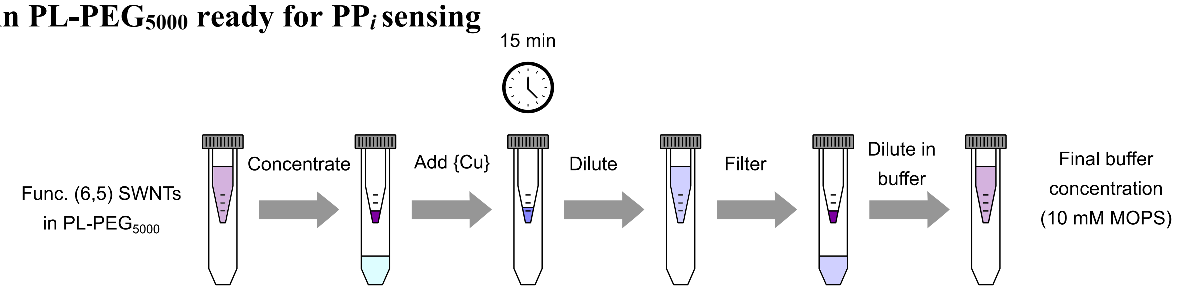

Supplementary Fig. 27 | Worflow of sample preparation. Sample preparation of functionalized (6,5) SWNTs dispersed in PL-PEG$_{5000}$. After an initial concentration step *via* spin-filtration the dispersion is incubated with {Cu} for 15 min. After incubation the dispersion is diluted with ultra-pure water, filtered and re-dispersed in 10 mM MOPS buffer.

Supplementary References

1 Niyogi, S. et al. Selective Aggregation of Single-Walled Carbon Nanotubes via Salt Addition. *J. Am. Chem. Soc.* **129**, 1898-1899 (2007).

2 Koh, B. & Cheng, W. Mechanisms of Carbon Nanotube Aggregation and the Reversion of Carbon Nanotube Aggregates in Aqueous Medium. *Langmuir* **30**, 10899-10909 (2014).

3 Yang, W., Chen, X., Su, H., Fang, W. & Zhang, Y. The fluorescence regulation mechanism of the paramagnetic metal in a biological HNO sensor. *Chem. Commun.* **51**, 9616-9619 (2015).

4 Rosenthal, J. & Lippard, S. J. Direct Detection of Nitroxyl in Aqueous Solution Using a Tripodal Copper(II) BODIPY Complex. *J. Am. Chem. Soc.* **132**, 5536-5537 (2010).

5 Su, H., Chen, X. & Fang, W. ON–OFF Mechanism of a Fluorescent Sensor for the Detection of Zn(II), Cd(II), and Cu(II)Transition Metal Ions. *Anal. Chem.* **86**, 891-899 (2014).

6 Shiraishi, T., Shiraki, T. & Nakashima, N. Substituent effects on the redox states of locally functionalized single-walled carbon nanotubes revealed by in situ photoluminescence spectroelectrochemistry. *Nanoscale* **9**, 16900-16907 (2017).

7 Bratsch, S. G. Standard Electrode Potentials and Temperature Coefficients in Water at 298.15 K. *J. Phys. Chem. Ref.* **18**, 1-21 (1989).

8 Chan, T. R., Hilgraf, R., Sharpless, K. B. & Fokin, V. V. Polytriazoles as Copper(I)-Stabilizing Ligands in Catalysis. *Org. Lett.* **6**, 2853-2855 (2004).

9 Miller, J. R., Calcaterra, L. T. & Closs, G. L. Intramolecular long-distance electron transfer in radical anions. The effects of free energy and solvent on the reaction rates. *J. Am. Chem. Soc.* **106**, 3047-3049 (1984).

10 Wulf, V. et al. Multicomponent System of Single-Walled Carbon Nanotubes Functionalized with a Melanin-Inspired Material for Optical Detection and Scavenging of Metals. *Adv. Funct. Mater.* **32**, 2209688 (2022).

11 Zhang, K., Xu, X.-H. & Qing, F.-L. Copper-Promoted Ritter-Type Trifluoro-ethoxylation of (Hetero)arenediazonium Tetrafluoroborates: A Method for the Preparation of Trifluoroethyl Imidates. *Eur. J. Org.* **2016**, 5088-5090 (2016).

12 Evrard, D., Lambert, F., Policar, C., Balland, V. & Limoges, B. Electrochemical Functionalization of Carbon Surfaces by Aromatic Azide or Alkyne Molecules: A Versatile Platform for Click Chemistry. *Chem. Eur. J.* **14**, 9286-9291 (2008).

13 Yu, B. et al. ortho-Substituted Aryldiazonium Design for the Defect Configuration-Controlled Photoluminescent Functionalization of Chiral Single-Walled Carbon Nanotubes. *ACS Nano* **16**, 21452-21461 (2022).

14 Schroll, P., Hari, D. P. & König, B. Photocatalytic Arylation of Alkenes, Alkynes and Enones with Diazonium Salts. *ChemistryOpen* **1**, 130-133 (2012).
