# Supplementary material for: Ratiometric fluorescent sensing of pyrophosphate with sp³-functionalized single-walled carbon nanotubes
Source: Nat Commun. 2024 Jan 24;15:706. doi: 10.1038/s41467-024-45052-1 (PMC10808354; doi:10.1038/s41467-024-45052-1)
Supplement: Supplementary file 4 — Source data [file 41467_2024_45052_MOESM4_ESM.zip › Raw_data_1H_NMR_2-ethynylbenzene diazonium tetrafluoroborate/pdata/1/email_jsd_ss_02_40010_1.pdf]

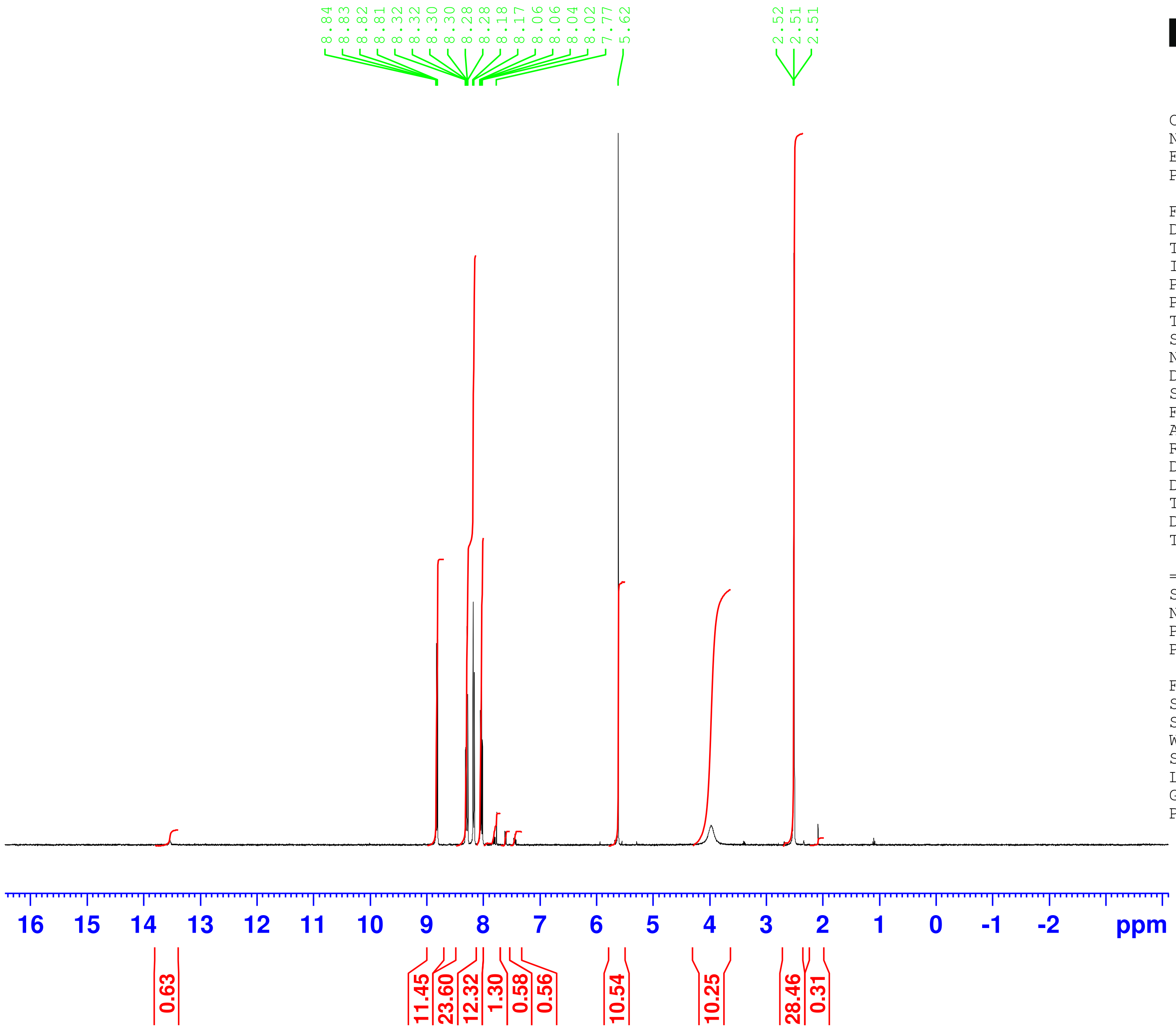

Current Data Parameters

NAME	jsd_ss_02
EXPNO	40010
PROCNO	1

F2 - Acquisition Parameters

Date_	20230327
Time	12.48
INSTRUM	spect
PROBHD	5 mm PABBO BB-
PULPROG	zg30
TD	65536
SOLVENT	DMSO
NS	8
DS	2
SWH	8223.685 Hz
FIDRES	0.125483 Hz
AQ	3.9845889 sec
RG	575
DW	60.800 usec
DE	6.50 usec
TE	294.6 K
D1	3.00000000 sec
TD0	1

======== CHANNEL f1 ========

SFO1	399.8924693 MHz
NUC1	1H
P1	9.00 usec
PLW1	25.00000000 W

F2 - Processing parameters

SI	65536
SF	399.8900000 MHz
WDW	EM
SSB	0
LB	0.30 Hz
GB	0
PC	20.00
