# Supplementary material for: Ratiometric fluorescent sensing of pyrophosphate with sp³-functionalized single-walled carbon nanotubes
Source: Nat Commun. 2024 Jan 24;15:706. doi: 10.1038/s41467-024-45052-1 (PMC10808354; doi:10.1038/s41467-024-45052-1)
Supplement: Supplementary file 4 — Source data [file 41467_2024_45052_MOESM4_ESM.zip › Raw_data_1H_NMR_4-ethynylbenzene diazonium tetrafluoroborate/pdata/1/Raw_data_1H_NMR_4-ethynylbenzene diazonium tetrafluoroborate.pdf]

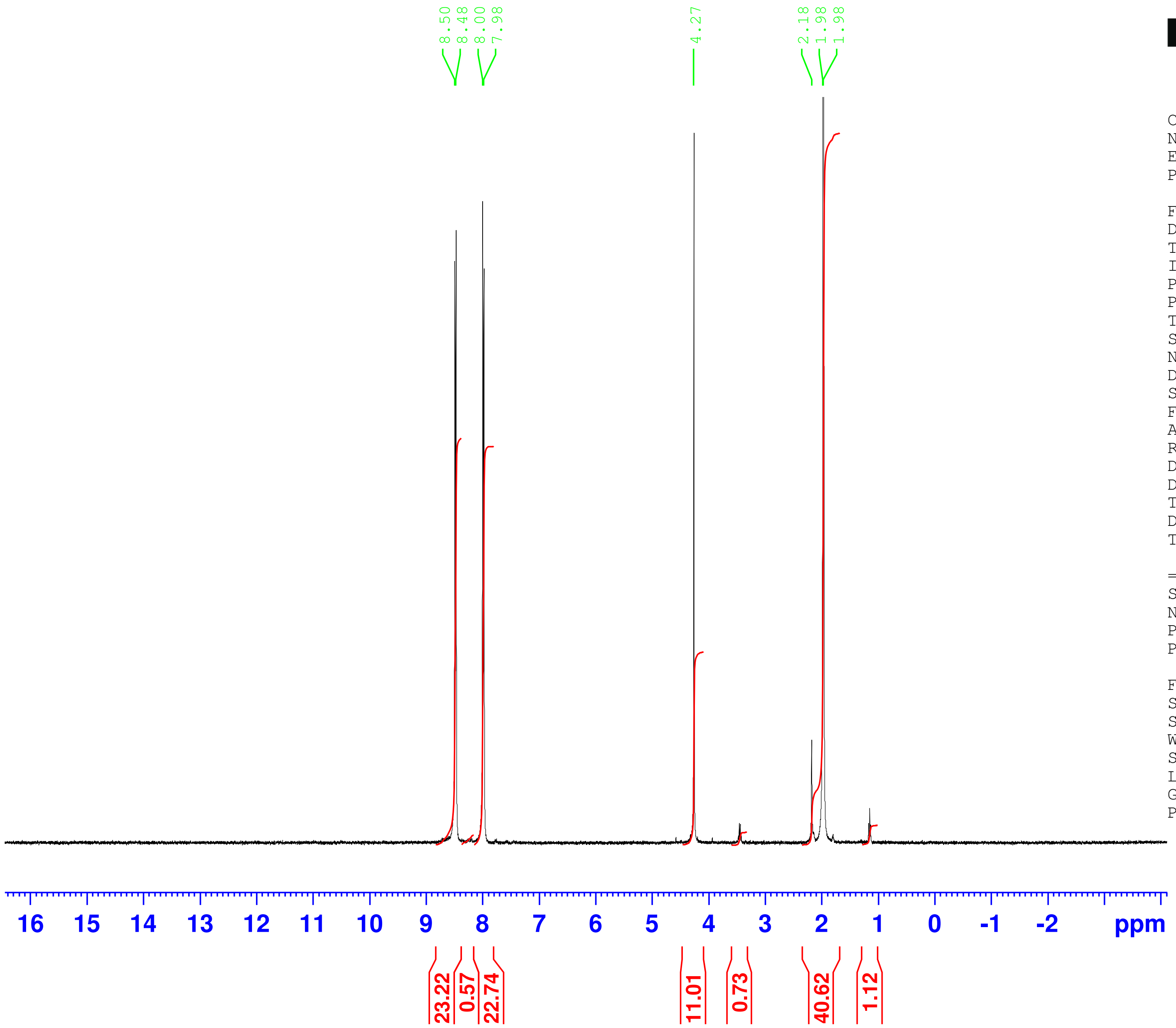

BRUKER

```
Current Data Parameters
NAME             jsd_ss_dz4
EXPNO                 40010
PROCNO                    1

F2 - Acquisition Parameters
Date_              20230327
Time                  10.29
INSTRUM               spect
PROBHD     5 mm PABBO BB-
PULPROG                zg30
TD                    65536
SOLVENT                CD3CN
NS                        8
DS                        2
SWH                8223.685 Hz
FIDRES             0.125483 Hz
AQ                3.9845889 sec
RG                      575
DW                   60.800 usec
DE                     6.50 usec
TE                    294.7 K
D1               3.00000000 sec
TD0                       1

======== CHANNEL f1 ========
SFO1            399.8924693 MHz
NUC1                     1H
P1                     9.00 usec
PLW1            25.00000000 W

F2 - Processing parameters
SI                    65536
SF              399.8900000 MHz
WDW                      EM
SSB      0
LB                     0.30 Hz
GB       0
PC                    20.00
```
