# Supplementary figures and images for: Ratiometric fluorescent sensing of pyrophosphate with sp³-functionalized single-walled carbon nanotubes
Source: Nat Commun. 2024 Jan 24;15:706. doi: 10.1038/s41467-024-45052-1 (PMC10808354; doi:10.1038/s41467-024-45052-1)

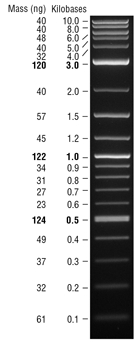

Supplement: Supplementary file 4 — Source data [file 41467_2024_45052_MOESM4_ESM.zip › Fig6f-NEB-1-kb-Plus-DNA-Ladder.jpg]

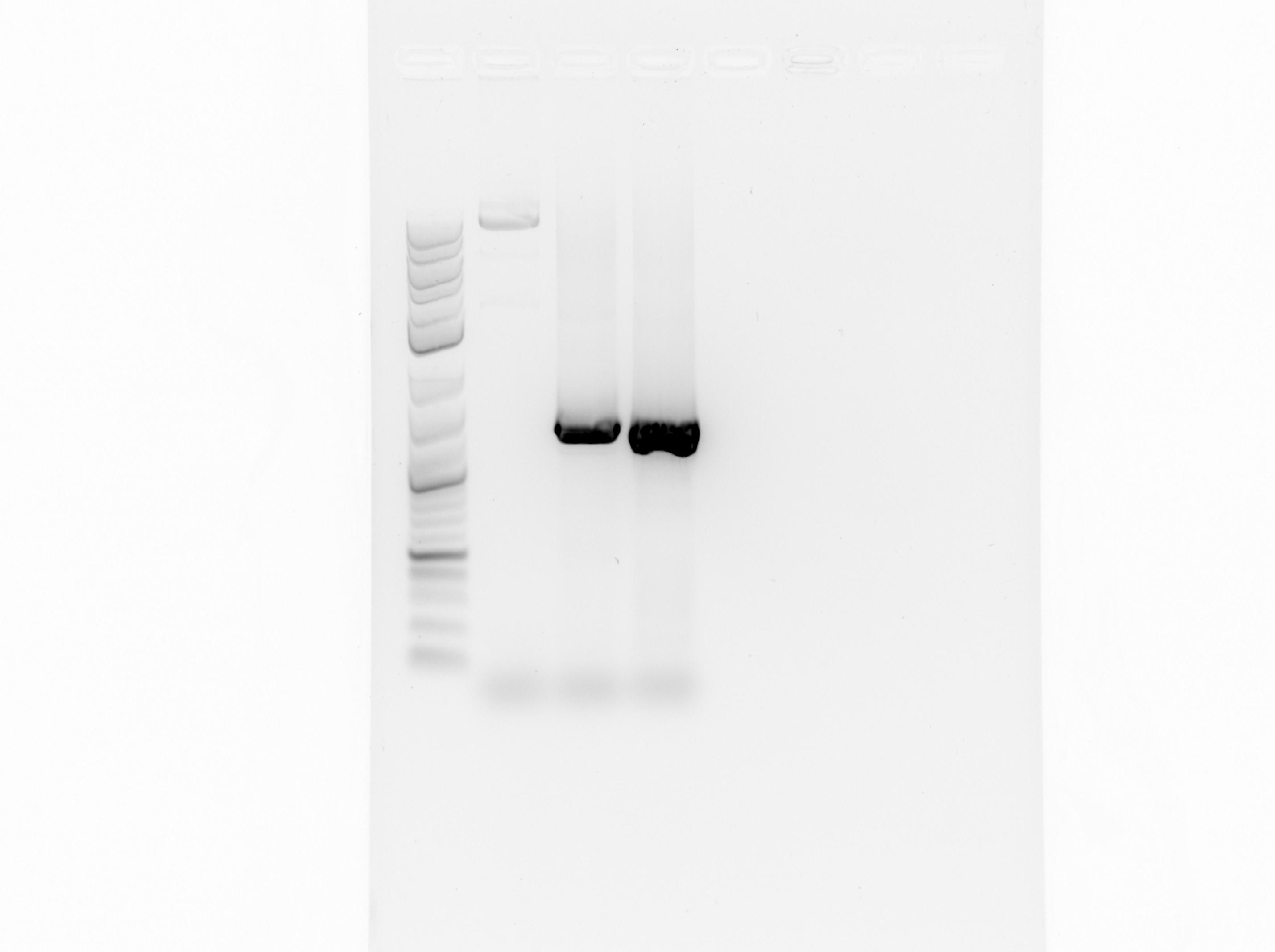

Supplement: Supplementary file 4 — Source data [file 41467_2024_45052_MOESM4_ESM.zip › Fig6f_PCR_agarose gel electrophoresis.tif]
